# Immediate vs. delayed endosseous integration of maxi implants: a torque removal animal study

**DOI:** 10.15171/joddd.2017.015

**Published:** 2017-06-21

**Authors:** Hanif Allahbakhshi, Fariborz Vafaee, Mehrdad Lotfazar, Ahmad Hasan Ahangary, Masoumeh Khoshhal, Farnoush Fotovat

**Affiliations:** ^1^Department of Prosthodontics, Faculty of Dentistry, Kashan University of Medical Sciences, Kashan, Iran; ^2^Department of Prosthodontics, Faculty of Dentistry, Hamadan University of Medical Sciences, Hamadan, Iran; ^3^Private Practice, Shiraz, Iran; ^4^Department of Periodontics, Faculty of Dentistry, Shiraz University of Medical Sciences, Shiraz, Iran; ^5^Department of Periodontics, Faculty of Dentistry, Hamadan University of Medical Sciences, Hamadan, Iran

**Keywords:** Denture, dental implant, dog, fixed, osseointegration, partial, torque

## Abstract

***Background.*** Delayed loading is one of the concerns in implant patients. Immediate loading can solve the problem and make patients more satisfied. The present study aimed to compare the removal torque of maxi implants under different loading (immediate and delayed) patterns.

***Methods.*** This split-mouth experimental study included 2 dogs. Impressions were made and then all the premolars were extracted under general anesthesia. After a three-month healing period, 3 implants were inserted in each quadrant (a total of 12 implants). Anterior and posterior implants (the case group) were splinted by an acrylic temporary bridge in order to make the middle implants (the control group) off the occlusion. The dogs were sacrificed after 6 weeks and bone blocks were submitted for removal torque test. Data were analyzed with ANOVA (P<0.05).

***Results.*** Mean torque values for the cases and control groups were 46.82±25.58 and 59.88±15.19, respectively (P=0.582; not significant).

***Conclusion.*** It may be concluded that immediate loading does not reduce the reverse torque values of maxi implants. This supports the advantages of immediate loading for maxi implants.

## Introduction

The high success rate and predictability of outcomes with the conventional delayed loading techniques encouraged the dental implant profession to revise the surgical and the prosthetic protocols towards early and immediate loading techniques. Currently immediate loading is considered a predictable treatment strategy in implant dentistry.^1,2^ Less trauma, high patient acceptance and comfort, decreased anxiety, reduced overall treatment time and superior esthetics are among the most important advantages of immediate loading.^3,4^

On the other hand, the application of mini implants (OsteoCare™, Slough, Berkshire, UK) appears to be of high benefit, especially in clinical situations where narrower fixture diameters are indicated. Mini implants are placed with a more conservative approach and loaded immediately. There is also no need for bone grafting. Maxi implants (OsteoCare™, Slough, Berkshire, UK) were then developed to combine the main advantages of mini and conventional implants. The goal was to allow easy insertion, predictable stability and immediate loading concurrently. Although the dental implant literature includes thorough discussions on the different aspects of conventional implant therapy, maxi implants (OsteoCare™, Slough, Berkshire, UK) and their advantages are yet to be investigated.^5^ Improved osseointegration is obtained, especially when implants are loaded immediately or early on compromised sites.^6^

Recent reviews of the literature conclude that moderately rough surfaces (Sa 1–2 mm) show stronger bone response in experimental investigations than smoother or rougher surfaces.^7-9^ Better clinical outcome, however, can only be documented under challenging conditions such as direct loading, grafted bone or when using short implants.^10^

The present study aimed to compare the reverse torque test of maxi implant in maxi implant 3.75 mm in width and 13 mm in length in immediate loading and delayed groups in dogs.

## Methods

This study was approved by the Ethics Committee of Shiraz University of Medical Sciences. Two mixed-breed, male dogs were randomly selected and primarily examined by a veterinarian to ensure there was no interfering factor such as diabetes, osteoporosis, etc. The dogs were kept in a fasting state for 12 hours before anesthesia to prevent nausea and vomiting during the course of the surgery. General anesthesia was provided by a veterinarian by administration of 2% acepormazine (0.5 mg/kg) and then nesdonal (17 mg/kg). Condensing silicon (Speedex Coltene, Coltène/Whaledent AG, Altstätten, Switzerland) impressions of the entire dentition were then made for both dogs to be a model for future reference in making temporary prostheses and clear stents.

**Figure 1:**
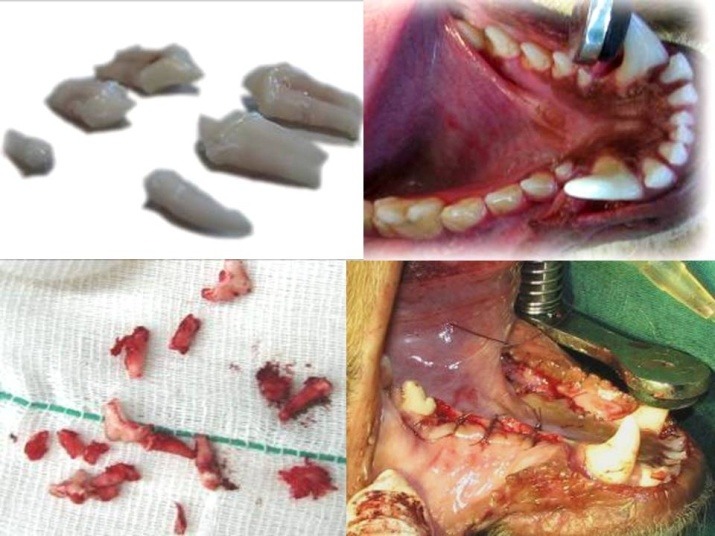
Surgical procedure of premolar extraction.

The teeth were extracted under sterile conditions. The first premolars were extracted by a simple rotational movement. The second and third premolars were vertically sectioned by a long knife-edged bur (SS White Burs, Inc., Lakewood, USA) and then extracted (Figure 1). Care was taken to save bone and make the surgical procedure as atraumatic as possible. Extraction sites were then sutured and diet was changed to a soft one for two weeks. Penicillin 200000 iu/kg was added to the diet for 5 days postoperatively to prevent infection.^11^ The periodontal status of the dogs was checked periodically due to the change in diet. Impressions were poured into dental stone casts and clear surgical stents were made accordingly using a vacuum machine and transparent sheets.

**Figure 2:**
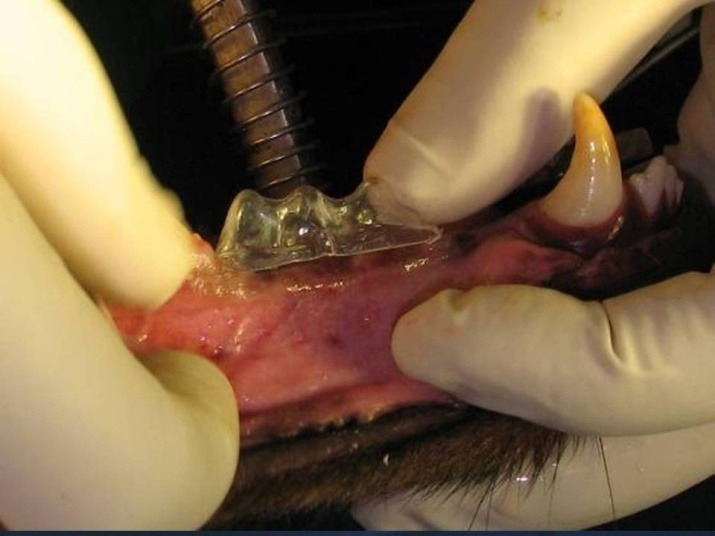
Clear surgical stents were made using a vacuum machine and transparent sheets.

**Figure 3:**
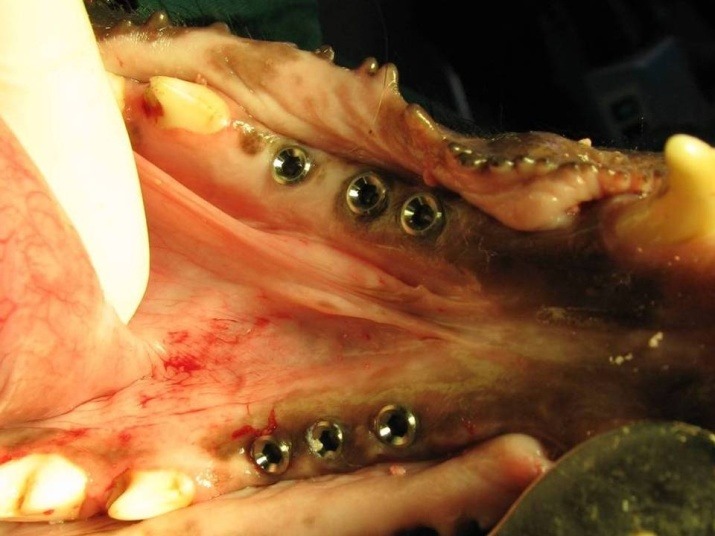
Implant placement in each premolar region.

After 3 months (the time needed for the healing of extraction sockets) the animals were given general anesthesia again and 3 tissue-level maxi implants (OsteoCare™, Slough, Berkshire, UK) measuring 3.75 mm in width and 13 mm in length were inserted in each premolar region of each dog (a total of 6 per dog) without osteotomy or flap (Figure 2). A surgical stent was used as an aid. Sufficient primary stability (32 and 40 Ncm)^12^ was assured by a torque wrench limit of 30 Ncm. Temporary prostheses were made (GC Tempron, GC Corporation, Shizuka, Japan) right after implantation (Figure 3). Using a direct technique, the first and the last implants were splinted. The middle implants were left embedded and hence off the occlusion (to serve as the control group). Also the tissue surface of the temporary prostheses was relieved over the middle implants using an acrylic resin polishing bur (SS White Burs, Inc. Lakewood, USA). A modified ridge lap was formed on the pontics. The soft diet was followed for two weeks postoperatively and penicillin 200000 iu/kg was added to diet for 5 days. Due to the high volume of torque-meter device and the need for histological evaluation, the dogs were sacrificed 6 weeks after surgery. Bone blocks containing the implants were removed from the jaw bone. Bone was removed using a diamond saw (Hager & Meisinger, Neuss, Germany) which cut with copious amount of water to decrease heat generation. Soft tissues overlying the bone were then reflected using a periosteal elevator (Hu-Friedy Europe, Zweigniederlassung Deutschland). The bone blocks were stored in 10% formalin and transferred to the laboratory.

Temporary acrylic prostheses were cut and removed and the healing abutments were detached. A diamond saw (Hager & Meisinger, Neuss, Germany) was used to cut the bone around each fixture. The blocks were fixed into polymethyl methacrylate self-curing acrylic resin (Acropars, Tehran, Iran) for reverse torque test. A coupling was made to fit the 2.2-mm internal diameter of the internal hex to the 9.6-mm external diameter of torque-meter. The torque was measured using a manual torque-meter in counter-clockwise motion and fixture withdraw torque values were recorded. Torque-meters were placed vertical to the long axis of fixtures and care was taken to avoid lateral forces.

**Figure 4:**
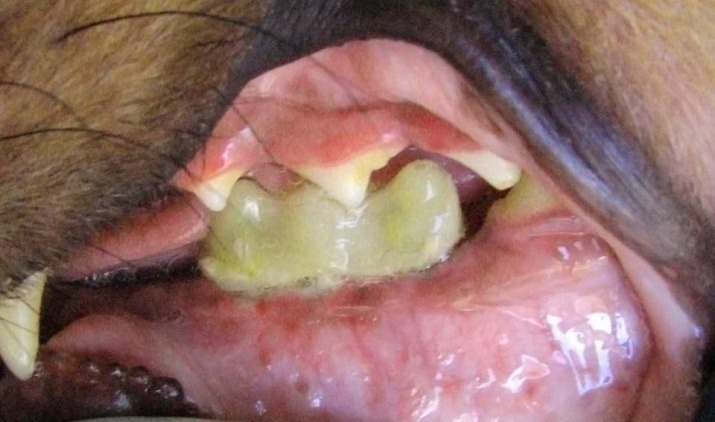
Temporary prostheses right after implantation.

For the RTT (removal torque test), wax cubes were prepared and poured with polymethyl methacrylate self-curing acrylic resin (Acropars, Tehran, Iran). The complex was then stored in formalin to avoid the adverse effect of polymerization heat on the bone‒fixture interface. The blocks were transferred to the laboratory for torque test.

ANOVA was used to analyze the mean differences of the removal torques (P<0.05).

## Results

The dogs were checked by a periodontist for periodontal status and by a prosthodontist for temporary bridge integrity and occlusion weekly and no problem was found during the test period until the last week, when one of the temporary bridges was broken (on the implant with the lowest removal torque test ).

Of 12 implants used in the present study, 8 were loaded immediately. One of the 8 immediately loaded implants failed to pass removal torque test with minimum torque (dog 2, group 2, left side) and the other 7 implants showed evidence of osseointegration (Table 1). Success rate was 87.5% in the immediately loaded group. Also 4 implants were placed without loading, for which a 100% success rate was observed. Overall success rate of the study implants was 91.6% (11 of 12 implants).

**Table 1:** Descriptive statistics of the mean removal torque of the groups (E1&E2: Implants loaded immediately, C: Implants maintained unloaded).

	**E1**		**C**	**E2**
**1L**	53.7		79.1	82.5
**1R**	75.8		47.7	50
**2L**	43.1		47.7	1.3
**2R**	34.4		65	33.8
**Mean**	51.75		59.88	41.90
**SD**	17.87		15.19	33.80

One of the temporary fixed partial dentures (FPD) showed fracture but it was not detached from the healing abutment (partially retained). Other 3 FPDs were actively in function throughout the experiment. The overall success rate was 75% (3 of 4) for the FPDs. The acrylic resin showed some abrasion.

Removal torque ranged from 1.3 Ncm to 83 Ncm. This value was only 1.3 Ncm for the failed implant. The overall success rate was 91.6%. The mean removal torques were 51.75%±17.87, 41.90±33.80 and 59.88±15.19 in groups 1 and 2 and the control, respectively.

**Figure 5:**
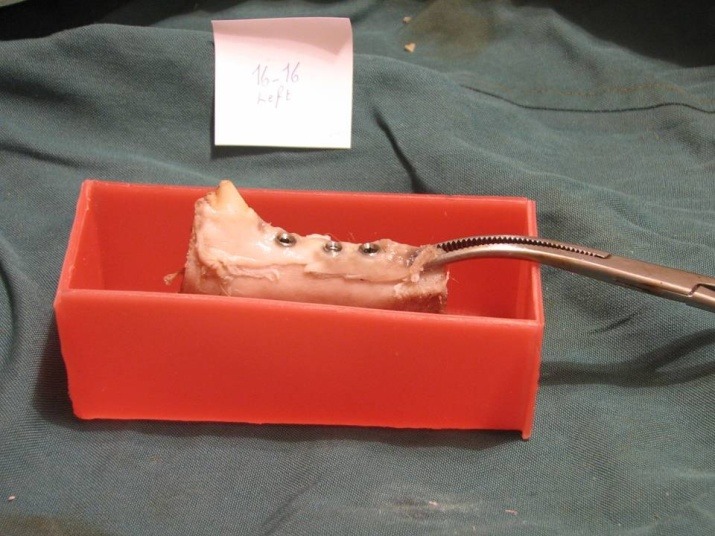
Bone blocks containing implants were removed from the jaw bone.

## Discussion

High success rate of immediate loading of implants led the profession to revise the surgical and prosthodontic protocols and expect a high success rate. Patients with immediately loaded restorations have the advantage of immediate rehabilitation of mastication.

An insertion torque of 32 to 40 Ncm is believed to be sufficient for a proper primary stability.^12^ For this reason, an insertion torque of 30 N was assured with the application of a torque wrench.

Implant failure is highly dependent on the implant type. While a high percentage of MTI mini-implants were lost in posterior mandible, standard implants were totally successful.^13,14^ Maxi implants apply a combination of mini-implant theories (autoadvance and autocondensing) and the width of standard implants. They are self-drilling, self-tapping tow-piece dental implants that can be used in different bone qualities; also they have a buttress thread form and GBA (grit-blasted and acid-etched) surface.^5^ The present findings indicate the success of maxi implants. The success rate was 87.5% in the immediately loaded group and 100% in the delayed loaded groups. These values are significantly higher than those of mini-implants and are comparable to the same values of conventional (standard) implants. Brunski^1^ and Lum et al^2^ reported 100% success in the control group and 100% failure in the immediate loading group. Zubery et al^14^ reported a 58% success rate in immediately loaded MTI Dentatus mini-implants and a 50% success rate in the control group. El-marssafy et al^15^ showed that the Osteocare’s Maxi Z one-piece, self-tapping self-drilling implant has a high success rate regarding initial stability and successful osseointegration. Acocella et al^16^ also presented data from a 3-year prospective study on immediately placed implants after tooth extractions in various clinical situations.^17^

Sagara^18^ and Piattelli^20^ reported 100% success rate in both immediately loaded and control groups. In two cases, the internal submerged implants were covered with soft tissue, which was indicative of the high biocompatibility of implant alloy. Sato et al^21^ stated that immediate loading might not inhibit osseointegration for smooth and rough implants in the late healing stages. However, Felice et al^22^ showed that there were more complications at immediate post-extractive implants when compared to delayed implants.

Resistance to reverse torque in implants with similar size, topography and design depends on the interfacial contact of fixture and bone.^23,24^ Reverse torque reflects the shear strength at the interface of implant and surrounding tissues. Of course, bone geometry and properties are also influential in reverse torque values.^25^

It has been shown that fixed partial dentures reduce the occlusal loads directed to the interface of implant and bone to the level of physiologic tolerance of bone.^26^ In the present study, one of the implants with mobile prosthesis was not osseointegrated. This indicates the importance of splinting and its effect on osseointegration. Sagara^18^ and Akagawa^27^ used fixed partial dentures; Piattelli^11^ and Corigliano^28^ used single crowns; and Akagawa^3^ used the abutment (no prosthesis) for loading.

Failure of temporary crowns was one of the main problems in similar studies. Different reinforcement techniques, including temporary crown with a single-strand wire, metal plate, collar and multiple wires, have been discussed in the literature. Multiple wire technique was applied in the present study because it has been widely used and accepted.^29^

Proper oral hygiene is mandatory in the course of healing of the immediately loaded implants.^18,19^ Emergence profiles in the present study were then adjusted to self-cleansing form using an acryl preparation bur.

The last and the most important consideration in the preparation of the single crowns was occlusion. Due to the needed occlusion of the crowns, they were prepared to be higher than the occlusal surface and then corrected to ideal occlusion. Due to the presence of airway tube, occlusal check was not possible during the experiment. The occlusion was then corrected with the addition of acrylic resin or the reduction of the premature contacts. In the present study the overall success rate of the implants was 75%, consistent to the findings of Emeka Nkante et al (71.4%).^30^

## Conclusion

Within the limitations of the present study, it might be concluded that immediate loading does not decrease reverse torque values of maxi implants. This supports the advantages of immediate loading for maxi implants but it requires further investigations to generalize to humans.

## Acknowledgments

The authors would like to thank the animal laboratory for the assistance provided during the experiments of this study.

## Funding

This study was supported by a fund from Shiraz University of Medical Sciences.

## Competing interests

The authors declare no competing interests with regards to the authorship and/or publication of this article.

## Ethics approval

The study was approved by the Ethics Committee of Shriaz University of Medical Sciences.
